# Epidemiology and outcomes in pediatric neuroblastoma: a US population-based study

**DOI:** 10.3389/fmed.2026.1861046

**Published:** 2026-10-15

**Authors:** Shruti Wadhwani, Nikita Wadhwani, Vignesh K. Nagesh, Izage Kianifar Aguilar, Behzad Amoozgar, Sindhu Avvari, Kousik Surya Sridharan, Aman Siddiqui, Urveesh Sharma, Olga Velichko, Kwangha Baek, Auda Auda, Valentino O. Baldeo, Tirth Bhavsar, Georgia Souza, Harman Antaal, Florchi Furman, Priya Rokade, Gopal Mondal, Pavan T. Vaddera, Divya Bhagavathi, Sumreen Nazly, Dessislava Papantoniou, Grace John, Dhanush K. Aruljothi, Reshma Radhakrishnan, Juilee V. Dongre

**Affiliations:** 1 Department of Internal Medicine, Hackensack Meridian Health, North Bergen, NJ, United States; 2 Department of Hematology and Oncology, John Theurer Cancer Center, Hackensack University Medical Center, Hackensack, NJ, United States; 3 Department Family Medicine, Hackensack Meridian Health, North Bergen, NJ, United States

**Keywords:** epidemiology, neuroblastoma, outcomes, pediatric, survival

## Abstract

**Purpose:**

Neuroblastoma is a heterogeneous pediatric malignancy with variable outcomes. This study aimed to evaluate survival outcomes and identify independent prognostic factors using a large population-based cohort.

**Methods:**

The Surveillance, Epidemiology, and End Results (SEER) database was used to identify 863 patients diagnosed with neuroblastoma between 2000 and 2017. Multivariable Cox proportional hazards regression was performed to assess predictors of overall and cancer-specific mortality.

**Results:**

The cohort included nearly equal numbers of males and females, with most patients diagnosed before 10 years of age. The majority of patients were non-Hispanic White, followed by Hispanic and non-Hispanic African American patients. In univariate analysis, race/ethnicity showed differences in mortality; however, these were not significant after multivariable adjustment. Increasing age at diagnosis was strongly associated with worse survival, with infants (<1 year) demonstrating the best outcomes and adolescents (10–19 years) the highest mortality risk. Distant metastatic disease was independently associated with increased mortality. Surgical resection was associated with improved overall survival, whereas chemotherapy was associated with higher mortality, likely reflecting confounding by indication due to treatment selection in high-risk disease.

**Conclusion:**

Age at diagnosis and disease stage are the strongest independent predictors of survival in pediatric neuroblastoma. Race/ethnicity was not independently associated with outcomes after adjustment. Observed associations between chemotherapy and mortality likely reflect underlying disease severity rather than treatment effect.

## Introduction

Neuroblastoma is a major cause of cancer-related morbidity and mortality in children. It is the most common extracranial solid tumor of infancy and early childhood and accounts for approximately 15% of pediatric cancer deaths. The incidence of neuroblastoma is estimated at 10.7 cases per one million children aged 0–14 years in the United States, although the true incidence may be underestimated because some tumors undergo spontaneous regression without clinical detection. The median age at diagnosis is approximately 23 months, with most cases diagnosed before 5 years of age. Neuroblastoma arises from primitive sympathetic neural crest cells and most commonly originates in the adrenal medulla, although primary tumors may also develop in the paraspinal sympathetic ganglia of the chest, abdomen, or pelvis (1–3).

Neuroblastoma exhibits remarkable clinical heterogeneity, ranging from spontaneous regression to aggressive metastatic disease with poor survival outcomes. Prognosis is influenced by multiple clinical and biological factors, including age at diagnosis, disease stage, histopathology, MYCN amplification, chromosomal abnormalities, and other molecular features (4–7). Patients are stratified into risk groups based on these characteristics to guide treatment intensity and predict outcomes. At diagnosis, approximately half of patients have metastatic disease, most commonly involving the bone marrow, bone, and lymph nodes, whereas central nervous system and lung metastases are uncommon. Bone marrow involvement, present in up to 80% of metastatic cases, may result in anemia and thrombocytopenia (8–10).

Accurate staging is essential for treatment planning and prognostication. Diagnostic evaluation includes cross-sectional imaging with computed tomography (CT) or magnetic resonance imaging (MRI), functional imaging with meta-iodobenzylguanidine (MIBG) scintigraphy, tumor biopsy for histopathologic confirmation, and molecular testing to identify prognostically important genetic alterations. Elevated urinary catecholamine metabolites, including vanillylmandelic acid (VMA), homovanillic acid (HVA), and dopamine, are detected in approximately 90% of patients and support the diagnosis. Treatment is individualized according to risk category and typically involves combinations of surgery, chemotherapy, radiation therapy, immunotherapy, targeted therapy, and, for high-risk disease, stem cell transplantation (11–13).

Despite substantial advances in risk stratification and multimodal therapy, outcomes remain poor for patients with high-risk neuroblastoma, and population-based data evaluating long-term prognostic factors remain limited. Therefore, this study used the Surveillance, Epidemiology, and End Results (SEER) database to characterize demographic and clinical features and identify independent predictors of overall and cancer-specific mortality among pediatric patients with neuroblastoma.

## Materials & methods

This population-based retrospective cohort study of pediatric neuroblastoma was conducted using the Surveillance, Epidemiology, and End Results (SEER) database, a comprehensive and widely used source of population-level cancer data. The SEER program, supported by the United States National Cancer Institute (NCI), collects information from 18 population-based cancer registries and covers approximately 28% of the U.S. population. The database includes detailed information on cancer incidence, patient demographics, clinicopathologic characteristics, treatment, and survival outcomes. Because SEER data are publicly available and contain de-identified patient information, institutional review board approval and informed consent were not required for this study.

## Inclusion and exclusion criteria

Neuroblastoma cases were identified from the SEER database according to the International Classification of Diseases for Oncology, Third Edition (ICD-O-3), using histology codes 9500 and 9490 with malignant behavior (/3). Primary sites were included within the ICD-O-3 primary site range C000-C809, consistent with the SEER pediatric neuroblastoma classification. The study included patients aged 0–19 years who were diagnosed between 2000 and 2017. Patients with missing information on age, race/ethnicity, or disease stage were excluded. After application of the inclusion and exclusion criteria, the final analytic cohort consisted of 863 patients.

## Study variables

Sociodemographic and tumor-related variables assessed in this study included age at diagnosis, gender, race/ethnicity (non-Hispanic White, non-Hispanic African American, Hispanic, and other), primary tumor site, tumor stage (localized, regional, or distant), area of residence, annual household income, surgical treatment, and treatment modalities (chemotherapy and radiotherapy). The primary outcomes were overall survival and cancer-specific survival. Overall mortality was defined as death from any cause during the follow-up period, whereas cancer-specific mortality was defined as death attributable to neuroblastoma.

## Statistical analysis

Statistical analyses were performed using Stata 18. Overall survival and cancer-specific survival were evaluated using Cox proportional hazards regression. Univariate Cox regression analyses were initially performed to examine the associations between individual demographic, clinical, tumor-related, and treatment characteristics and mortality. Multivariable Cox regression models were then constructed to identify factors independently associated with overall and cancer-specific mortality. Covariate selection for the multivariable models was guided by clinical relevance, established prognostic importance in neuroblastoma, and potential confounding. Variables considered clinically relevant, including age at diagnosis, sex, race/ethnicity, disease stage, primary tumor site, and treatment-related variables, were considered for inclusion in the adjusted models.

Hazard ratios (HRs) greater than 1 indicated an increased hazard of mortality, whereas HRs less than 1 indicated a decreased hazard. Results are presented as HRs with corresponding 95% confidence intervals (CIs). All statistical tests were two-sided, and a *p*-value <0.05 was considered statistically significant. The proportional hazards assumption underlying the Cox regression models was not formally tested and is acknowledged as a limitation of the study. Given the number of observed events relative to the number of covariates included in the multivariable models, potential model instability and overfitting were also considered when interpreting the adjusted estimates.

## Results

In this population-based cohort of 863 pediatric patients with neuroblastoma (Table 1), we evaluated demographic, clinical, and treatment-related characteristics and their associations with overall and cancer-specific survival using SEER data from 2000 to 2017. The cohort demonstrated a near-equal gender distribution and was predominantly composed of children aged 1–9 years (60.25%). Non-Hispanic White patients constituted the majority (55.04%), followed by Hispanic (25.25%) and non-Hispanic African American (11.59%) patients. Most tumors were located in the peripheral or autonomic nervous system, with approximately one-third of patients presenting with distant metastatic disease (32.79%). Surgical resection was performed in nearly all patients (97.7%), while 58.52% received chemotherapy and 15.53% received radiotherapy.

**Table 1:** Demographic and clinicopathologic characteristics of pediatric neuroblastoma diagnosed between 2000 and 2017 in the United States.

Characteristics	Number of patients [total sample size - 863]	Percentage
Gender		
Female	430	49.83
Male	433	50.17
Age at diagnosis in years		
<1	296	34.3
01–09	520	60.25
10–19	47	5.45
Ethnoracial identity		
Non-Hispanic Caucasian	475	55.04
Non-Hispanic African-American	100	11.59
Hispanic	218	25.26
Other	70	8.11
Tumor site		
Nervous system	15	1.74
Connective, subcutaneous, other soft tissues of abdomen and gastrointestinal tract	60	6.95
Connective, subcutaneous, other soft tissues of thorax, trunk and lung	106	12.28
Connective, subcutaneous, other soft tissues of the head, neck, and face	30	3.48
Connective, subcutaneous, other soft tissues of the limbs and pelvis	38	4.4
Kidney, renal pelvis and ovary	34	3.94
Brain	37	4.29
Connective, subcutaneous, and other soft tissues, overlap	30	3.48
Spinal cord	40	4.63
Peripheral & autonomic nervous system of head, face, neck	43	4.98
Peripheral & autonomic nervous system of limbs and hip	47	5.45
Peripheral & autonomic nervous system of trunk/thorax	190	22.02
Peripheral & autonomic nervous system of the abdomen	120	13.9
Overlap of peripheral and autonomic nervous system	73	8.46
Tumor stage		
Localized	271	31.4
Regional	309	35.81
Distant	283	32.79
Area of residence		
Counties in metropolitan areas of 1 million persons	542	62.8
Counties in metropolitan areas of 250,000 to 1 million persons	189	21.9
Counties in metropolitan areas of 250,000 persons	68	7.88
Nonmetropolitan counties adjacent to a metropolitan area	40	4.63
Nonmetropolitan counties not adjacent to a metropolitan area	24	2.78
Income per year		
< $45,000	20	2.32
$45,000–54,999	81	9.39
$55,000–64,999	141	16.34
$65,000–74,999	251	29.08
$75,000+	370	42.87
Radiation		
No	729	84.47
Yes	134	15.53
Chemotherapy		
No	358	41.48
Yes	505	58.52
Surgery		
No	20	2.32
Yes	843	97.68
Year of diagnosis		
2000	43	4.98
2001	56	6.49
2002	46	5.33
2003	35	4.06
2004	48	5.56
2005	58	6.72
2006	36	4.17
2007	61	7.07
2008	41	4.75
2009	56	6.49
2010	45	5.21
2011	60	6.95
2012	38	4.4
2013	54	6.26
2014	55	6.37
2015	55	6.37
2016	41	4.75
2017	35	4.06

Univariate Cox regression analysis (Table 2) identified multiple factors significantly associated with survival outcomes. Age at diagnosis demonstrated a strong stepwise association with mortality. Compared with infants (<1 year), children aged 1–9 years had significantly increased overall mortality (HR 4.34, 95% CI 2.43–7.76) and cancer-specific mortality (HR 5.10, 95% CI 2.65–9.83), while those aged 10–19 years had even higher risks (HR 7.08 and 7.23, respectively). Non-Hispanic African American patients experienced higher overall (HR 1.86) and cancer-specific mortality (HR 1.79) compared with non-Hispanic White patients. Tumor stage was a major determinant of outcome, with distant metastatic disease associated with markedly increased mortality (overall HR 12.68; cancer-specific HR 15.37). In contrast, tumors arising in connective tissues of the thorax, trunk, and lung were associated with lower mortality risk.

**Table 2:** Univariate analyses of factors affecting all-cause mortality and neuroblastoma related mortality among US children and adolescents diagnosed between 2000 and 2017.

Characteristics	Overall mortality; crude proportional hazard ratio (95% confidence interval)	Cancer-specific mortality; crude proportional hazard ratio (95% confidence interval)
Gender		
Female	1 (reference)	1 (reference)
Male	1.41 (0.98–2.01)	1.28 (0.87–1.87)
Age at diagnosis in years		
<1	1 (reference)	1 (reference)
1–9	4.34 (2.43–7.76) **	5.1 (2.65–9.83) **
10–19	7.08 (3.33–15.06) **	7.23 (3.07–17.04) **
Ethnoracial identity		
Non-Hispanic Caucasian	1 (reference)	1 (reference)
Non-Hispanic African-American	1.86 (1.17–2.96) **	1.79 (1.08–2.98) *
Hispanic	0.76 (0.47–1.23)	0.79 (0.48–1.33)
Other	0.95 (0.47–1.91)	1.09 (0.54–2.21)
Tumor site		
Nervous system	1 (reference)	1 (reference)
Connective, subcutaneous, other soft tissues of abdomen and gastrointestinal tract	0.764 (0.25–2.37)	0.7 (0.22–2.21)
Connective, subcutaneous, other soft tissues of thorax, trunk and lung	0.15 (0.04–0.56) **	0.11 (0.02–0.39) **
Connective, subcutaneous, other soft tissues of the head, neck, and face	0.31 (.07–1.39)	0.1 (0.01–0.93) *
Connective, subcutaneous, other soft tissues of the limbs and pelvis	0.25 (0.06–1.14)	0.17 (0.05–0.95) *
Kidney, renal pelvis and ovary	0.29 (0.06–1.29)	0.19 (0.04–1.05)
Brain	1.27 (0.41–4.02)	0.94 (0.28–3.11)
Connective, subcutaneous, and other soft tissues, overlap	0.38 (0.08–1.68)	0.38 (0.08–1.69)
Spinal cord	0.43 (0.12–1.59)	0.34 (0.08–1.38)
Peripheral & autonomic nervous system of head, face, neck	0.145 (0.03–0.79)	0.15 (0.03–0.79) *
Peripheral & autonomic nervous system of limbs and hip	0.43 (0.12–1.53)	0.36 (0.09–1.35)
Peripheral & autonomic nervous system of trunk/thorax	0.248 (0.08–0.75)	0.22 (0.07–0.66)
Peripheral & autonomic nervous system of the abdomen	1.02 (0.36–2.88)	0.97 (0.34–2.75)
Overlap of peripheral and autonomic nervous system	0.67 (0.22–2.04)	0.62 (0.2–1.91)
Tumor stage		
Localized	1 (reference)	1 (reference)
Regional	2.22 (0.98–5.01)	2.26 (0.89–5.79) **
Distant	12.68 (6.15–26.11) **	15.37 (6.71–35.2) **
Area of residence		
Counties in metropolitan areas of 1 million persons	1 (reference)	1 (reference)
Counties in metropolitan areas of 250,000 to 1 million persons	1.36 (0.89–2.05)	1.21 (0.77–1.9)
Counties in metropolitan areas of 250,000 persons	0.65 (0.28–1.49)	0.71 (0.31–1.62)
Nonmetropolitan counties adjacent to a metropolitan area	1.49 (0.71–3.08)	1.21 (0.53–2.8)
Nonmetropolitan counties not adjacent to a metropolitan area	1.09 (0.35–3.49)	0.8 (0.19–3.28)
Income per year		
< $45,000	1 (reference)	1 (reference)
$45,000–54,999	0.83 (0.23–2.98)	0.69 (0.19–2.53)
$55,000–64,999	0.92 (0.27–3.07)	0.66 (0.19–2.28)
$65,000–74,999	0.69 (0.21–2.29)	0.65 (0.19–2.15)
$75,000+	0.96 (0.29–3.05)	0.88 (0.28–2.83)
Chemotherapy		
No	1 (reference)	1 (reference)
Yes	7.47 (4.02–13.89) **	10.55 (4.91–22.72) **
Radiation		
No	1 (reference)	1 (reference)
Yes	3.91 (2.73–5.63) **	4.41 (3.00–6.48) **
Surgery		
No	1 (reference)	1 (reference)
Yes	0.26 (0.13–0.54) **	0.375 (0.15–0.92) *

*p < .05.

**p < .01.

Treatment variables in univariate analysis (Table 2) showed that patients receiving chemotherapy (overall HR 7.47; cancer-specific HR 10.55) and radiotherapy (HR 3.91 and 4.41, respectively) had significantly higher mortality, while surgical resection was associated with improved survival (overall HR 0.26; cancer-specific HR 0.38). These associations likely reflect underlying disease severity and treatment selection bias rather than direct causal effects.

Multivariable Cox regression analysis (Table 3) confirmed several independent prognostic factors. Male gender was associated with increased overall (HR 2.02, 95% CI 1.31–3.11) and cancer-specific mortality (HR 1.64, 95% CI 1.04–2.61). Age remained the strongest predictor of outcome, with children aged 1–9 years (HR 4.78 and 5.35) and 10–19 years (HR 11.36 and 12.21) showing progressively higher risks compared with infants. Distant metastatic disease remained a powerful independent predictor of mortality (overall HR 6.67; cancer-specific HR 6.64). Surgical resection was independently associated with improved overall survival (HR 0.30, 95% CI 0.13–0.73).

**Table 3:** Multivariate Cox proportional hazard analyses of factors affecting all-cause mortality and neuroblastoma related mortality among US children and adolescents diagnosed between 2000 and 2017.

Characteristics	Overall mortality; adjusted proportional hazard ratio (95% confidence interval)	Cancer-specific mortality; adjusted proportional hazard ratio (95% confidence interval)
Gender		
Female	1 (reference)	1 (reference)
Male	2.02 (1.31–3.11) **	1.64 (1.04–2.61) *
Age at diagnosis in years		
<1	1 (reference)	1 (reference)
1–9	4.78 (2.48–9.17) **	5.35 (2.58–11.06) **
10–19	11.36 (4.69–27.51) **	12.21 (4.56–32.63) **
Ethnoracial identity		
Non-Hispanic Caucasian	1 (reference)	1 (reference)
Non-Hispanic African-American	1.51 (0.86–2.67)	1.28 (0.69–2.36)
Hispanic	0.86 (0.49–1.49)	1.002 (0.56–1.79)
Other	0.87 (0.402–1.87)	0.97 (0.44–2.15)
Tumor site		
Nervous system	1 (reference)	1 (reference)
Connective, subcutaneous, other soft tissues of abdomen and gastrointestinal tract	0.57 (0.16–2.02)	0.56 (0.16–2.07)
Connective, subcutaneous, other soft tissues of thorax, trunk and lung	0.20 (0.05-.81) *	0.24 (0.06–0.98) *
Connective, subcutaneous, other soft tissues of the head, neck, and face	0.69 (0.14–3.55)	0.301 (0.03–3.02)
Connective, subcutaneous, other soft tissues of the limbs and pelvis	0.32 (0.06–1.63)	0.26 (0.04–1.64)
Kidney, renal pelvis and ovary	0.29 (0.06–1.46)	0.22 (0.04–1.31)
Brain	0.78 (0.22–2.83)	0.702 (0.18–2.73)
Connective, subcutaneous, and other soft tissues, overlap	0.17 (0.02–0.92) *	0.27 (0.05–1.42)
Spinal cord	0.72 (0.17–3.02)	0.79 (0.17–3.68)
Peripheral & autonomic nervous system of head, face, neck	0.35 (0.06–2.12)	0.45 (0.07–2.78)
Peripheral & autonomic nervous system of limbs and hip	0.45 (0.11–1.78)	0.42 (0.09–1.78)
Peripheral & autonomic nervous system of trunk/thorax	0.29 (0.09–0.98) *	0.31 (0.08–1.11)
Peripheral & autonomic nervous system of the abdomen	0.88 (0.28–2.81)	0.96 (0.29–3.17)
Overlap of peripheral and autonomic nervous system	0.69 (0.204–2.31)	0.71 (0.21–2.48)
Tumor stage		
Localized	1 (reference)	1 (reference)
Regional	1.92 (0.81–4.57)	1.76 (0.65–4.75)
Distant	6.67 (2.92–15.26) **	6.64 (2.61–16.89) **
Area of residence		
Counties in metropolitan areas of 1 million persons	1 (reference)	1 (reference)
Counties in metropolitan areas of 250,000 to 1 million persons	1.49 (0.89–2.48)	1.38 (0.79–2.39)
Counties in metropolitan areas of 250,000 persons	0.82 (0.29–2.31)	0.94 (0.32–2.71)
Nonmetropolitan counties adjacent to a metropolitan area	1.53 (0.55–4.24)	1.24 (0.4–3.84)
Nonmetropolitan counties not adjacent to a metropolitan area	1.104 (0.26–4.67)	0.82 (0.15–4.34)
Income per year		
< $45,000	1 (reference)	1 (reference)
$45,000–54,999	0.59 (0.14–2.48)	0.63 (0.14–2.82)
$55,000–64,999	0.82 (0.19–3.42)	0.62 (0.14–2.72)
$65,000–74,999	0.66 (0.15–2.88)	0.57 (0.13–2.59)
$75,000+	0.69 (0.16–3.09)	0.6 (0.13–2.801)
Chemotherapy		
No	1 (reference)	1 (reference)
Yes	3.09 (1.49–6.42) **	4 (1.67–9.6) **
Radiation		
No	1 (reference)	1 (reference)
Yes	1.29 (0.66–2.57)	1.62 (0.81–3.25)
Surgery		
No	1 (reference)	1 (reference)
Yes	0.303 (0.13–0.73) **	0.48 (0.16–1.39)

*p < .05.

**p < .01.

In contrast, ethnoracial differences observed in univariate analysis were no longer statistically significant in multivariable models (Table 3), suggesting that crude disparities may be explained by differences in disease stage, socioeconomic factors, or access to care rather than independent biological differences.

Tumor location also retained prognostic significance in adjusted analyses, with tumors arising in connective tissues of the thorax, trunk, and lung associated with reduced mortality (overall HR 0.20; cancer-specific HR 0.24). These findings may reflect differences in tumor biology, stage distribution, or surgical resectability across anatomical sites.

A key finding of this study is the association between chemotherapy and increased mortality in both univariate and multivariable models (Table 3: HR 3.09 for overall mortality; HR 4.00 for cancer-specific mortality). However, this finding must be interpreted with caution due to substantial confounding by indication. Chemotherapy is preferentially administered to patients with high-risk disease, including metastatic tumors and unfavorable biological features. Because SEER lacks essential neuroblastoma-specific prognostic variables such as MYCN amplification, DNA ploidy, histopathologic classification (INPC/Shimada), and segmental chromosomal aberrations, full adjustment for disease severity is not possible. Therefore, this association does not imply a harmful effect of chemotherapy but rather reflects underlying tumor aggressiveness.

Similarly, radiotherapy was associated with increased mortality, likely reflecting its use in refractory or high-risk disease settings. Surgical resection, in contrast, remained associated with improved survival, particularly overall survival, although this may partially reflect selection of patients with more favorable disease biology.

Several limitations should be considered. SEER does not include several critical neuroblastoma-specific risk stratification variables, including MYCN amplification status, DNA ploidy, histopathologic classification, and chromosomal abnormalities, limiting alignment with contemporary International Neuroblastoma Risk Group classification and potentially resulting in residual confounding. SEER staging is limited to broad categories of localized, regional, and distant disease and therefore lacks the granularity of contemporary neuroblastoma staging systems. Treatment information in SEER does not capture the timing, intensity, specific regimen, treatment response, or rationale for treatment selection, limiting assessment of treatment effects and introducing the potential for residual confounding by indication. The study period from 2000 to 2017 encompassed substantial changes in neuroblastoma diagnosis, risk stratification, and treatment, and year of diagnosis was not incorporated into the multivariable models, potentially introducing temporal confounding and heterogeneity in outcomes. Although Cox proportional hazards regression was used to evaluate time-to-event outcomes, the proportional hazards assumption was not formally tested, which represents a methodological limitation when interpreting the reported hazard ratios. In addition, the number of observed events relative to the number of covariates included in the multivariable models may raise concerns regarding model stability and potential overfitting. The observed association between chemotherapy and mortality may be substantially influenced by confounding by indication, as SEER does not capture several key clinical and molecular factors that determine treatment selection and disease risk. Finally, the retrospective observational design and reliance on registry data preclude definitive causal inference, particularly regarding treatment-related associations.

Despite these limitations, this study leverages a large, population-based cohort (Table 1) and provides robust real-world estimates of prognostic factors in pediatric neuroblastoma. The consistency of findings across univariate (Table 2) and multivariable (Table 3) analyses strengthens the observed associations, particularly for age, stage, and tumor location. In conclusion, analysis of SEER data demonstrates that male gender, older age at diagnosis, and distant metastatic disease are independently associated with increased mortality, while surgical resection is associated with improved survival. Observed associations with chemotherapy and radiotherapy are most consistent with confounding by indication. These findings highlight the importance of integrating molecular and clinical risk factors in future population-based neuroblastoma research to improve prognostic precision.

## Discussion

In this population-based analysis of pediatric neuroblastoma using SEER data (2000–2017), we identified key clinical and demographic predictors of survival outcomes. Overall, age at diagnosis and disease stage emerged as the most consistent determinants of mortality, while treatment-related associations were strongly influenced by underlying disease severity and selection effects.

A major finding of this study is the strong prognostic impact of age at diagnosis. Increasing age was associated with progressively worse survival, with infants demonstrating the most favorable outcomes and adolescents exhibiting the highest mortality risk. This age-dependent gradient is consistent with established neuroblastoma biology, in which infant tumors are more likely to exhibit favorable genetic features and spontaneous regression, whereas older children are more likely to present with biologically aggressive disease. These observations align with contemporary neuroblastoma risk classification systems that identify older age as a key adverse prognostic factor (14, 15).

Disease stage also remained a dominant predictor of outcome, with metastatic disease conferring substantially worse survival. This finding reinforces the persistent clinical challenge of high-risk neuroblastoma despite advances in multimodal therapy and underscores the importance of early detection and accurate staging (16).

Male gender was independently associated with poorer survival outcomes, although the biological mechanisms underlying this association remain uncertain and may reflect differences in tumor biology, immune response, or unmeasured social and healthcare-related factors.

Tumor location demonstrated additional prognostic relevance, suggesting that anatomical site may influence outcomes through differences in tumor biology and feasibility of complete surgical resection. Tumors in more surgically accessible regions appeared to be associated with more favorable outcomes.

Treatment-related findings require cautious interpretation. Although chemotherapy and radiotherapy were associated with higher mortality, these associations are most plausibly explained by confounding by indication, as these therapies are preferentially administered to patients with advanced or biologically high-risk disease. Importantly, SEER lacks key neuroblastoma-specific prognostic variables, including MYCN amplification, DNA ploidy, INPC/Shimada histopathology, and segmental chromosomal aberrations, limiting adequate adjustment for tumor biology (17, 18). Therefore, these associations should not be interpreted as evidence of treatment-related harm.

Similarly, the observed survival advantage associated with surgical resection likely reflects both therapeutic benefit in appropriately selected patients and selection bias toward patients with more localized or resectable disease. The absence of granular treatment data, including timing, intensity, and response to therapy, further limits causal inference.

Ethnoracial differences observed in univariate analyses were not maintained after multivariable adjustment, suggesting that apparent disparities are largely explained by clinical and socioeconomic factors rather than independent biological differences (19–24). However, limited representation of some racial groups may reduce statistical power to detect subtle differences.

Several methodological limitations should be acknowledged. The Cox proportional hazards model assumes proportional hazards (constant hazard ratios over time), and this assumption was not formally tested. In addition, the study spans a long period (2000–2017), during which neuroblastoma diagnostic and treatment strategies evolved substantially, and year of diagnosis was not included in multivariable models, introducing potential temporal confounding.

The SEER database also lacks essential molecular and biological markers central to modern neuroblastoma risk stratification, including MYCN amplification, DNA ploidy, INPC/Shimada classification, and segmental chromosomal aberrations such as 1p deletion, 11q loss, and 17q gain. Furthermore, SEER staging does not correspond to the International Neuroblastoma Risk Group (INRG) system, limiting clinical granularity. The absence of key neuroblastoma-specific variables in SEER limits comprehensive risk stratification and may contribute to residual confounding by underlying tumor biology. Therefore, our findings should be interpreted as population-level associations rather than as a substitute for contemporary molecular and integrated neuroblastoma risk classification.

Finally, an important limitation is the absence of external validation using an independent real-world cohort. Although SEER provides a large and nationally representative dataset, lack of external validation limits generalizability. Future studies incorporating institutional or multi-center datasets would strengthen the robustness of these findings.

Despite these limitations, the large sample size and population-based design of SEER provide meaningful epidemiological insight into prognostic factors in pediatric neuroblastoma.

## Conclusions

In conclusion, this retrospective population-based cohort study provides real-world evidence on survival outcomes in children and adolescents with primary neuroblastoma in the United States. Age at diagnosis and disease stage were the strongest independent predictors of mortality, with infants demonstrating the most favorable outcomes and increasing age, particularly adolescence, associated with progressively worse survival. Patients presenting with distant metastases also showed markedly poorer outcomes. Surgical resection was associated with improved overall survival, likely reflecting both therapeutic benefit and selection of patients with more localized or resectable disease. In contrast, the observed association between chemotherapy and increased mortality is most consistent with confounding by indication, as treatment is more frequently administered to patients with advanced or biologically high-risk disease.

Race and ethnicity were not independently associated with survival after multivariable adjustment. These findings underscore the dominant role of disease biology and extent in determining outcomes and highlight the limitations of administrative datasets that lack molecular and genomic risk stratification variables. Further studies incorporating detailed molecular, genomic, and treatment-intensity data, ideally with external validation cohorts, are needed to refine prognostic modeling and improve risk-adapted management in pediatric neuroblastoma.

## Data Availability

The datasets presented in this study can be found in online repositories. The names of the repository/repositories and accession number(s) can be found below: https://seer.cancer.gov/data/.
